# Functional versatility of Zur in metal homeostasis, motility, biofilm formation, and stress resistance in *Yersinia pseudotuberculosis*

**DOI:** 10.1128/spectrum.03756-23

**Published:** 2024-03-27

**Authors:** Yanchao Gu, Yongde Liu, Wei Mao, Ying Peng, Xiaoru Han, Han Jin, Jingling Xu, Liyang Chang, Yixin Hou, Xihui Shen, Xingyu Liu, Yantao Yang

**Affiliations:** 1 State Key Laboratory for Crop Stress Resistance and High-Efficiency Production, Shaanxi Key Laboratory of Agricultural and Environmental Microbiology, College of Life Sciences, Northwest A&F University, Yangling, China; 2 Qingyang Longfeng Sponge City Construction Management and Operation Co., Ltd, Qingyang, China; 3 College of Enology, Northwest A&F University, Yangling, China; 4 General Research Institute for Nonferrous Metals, Beijing, China; Rutgers New Jersey Medical School, Newark, New Jersey, USA

**Keywords:** Zur, transcriptomics, motility, stress resistance, biofilm formation

## Abstract

Zur (zinc uptake regulator) is a significant member of the Fur (ferric uptake regulator) superfamily, which is widely distributed in bacteria. Zur plays crucial roles in zinc homeostasis and influences cell development and environmental adaptation in various species. *Yersinia pseudotuberculosis* is a Gram-negative enteric that pathogen usually serves as a model organism in pathogenicity studies. The regulatory effects of Zur on the zinc transporter ZnuABC and the protein secretion system T6SS have been documented in *Y. pseudotuberculosis*. In this study, a comparative transcriptomics analysis between a ∆*zur* mutant and the wild-type (WT) strain of *Y. pseudotuberculosis* was conducted using RNA-seq. This analysis revealed global regulation by Zur across multiple functional categories, including membrane transport, cell motility, and molecular and energy metabolism. Additionally, Zur mediates the homeostasis not only of zinc but also ferric and magnesium *in vivo*. There was a notable decrease in 35 flagellar biosynthesis and assembly-related genes, leading to reduced swimming motility in the ∆*zur* mutant strain. Furthermore, Zur upregulated multiple simple sugar and oligopeptide transport system genes by directly binding to their promoters. The absence of Zur inhibited biofilm formation as well as reduced resistance to chloramphenicol and acidic stress. This study illustrates the comprehensive regulatory functions of Zur, emphasizing its importance in stress resistance and pathogenicity in *Y. pseudotuberculosis*.

**IMPORTANCE:**

Bacteria encounter diverse stresses in the environment and possess essential regulators to modulate the expression of genes in responding to the stresses for better fitness and survival. Zur (zinc uptake regulator) plays a vital role in zinc homeostasis. Studies of Zur from multiple species reviewed that it influences cell development, stress resistance, and virulence of bacteria. *Y. pseudotuberculosis* is an enteric pathogen that serves a model organism in the study of pathogenicity, virulence factors, and mechanism of environmental adaptation. In this study, transcriptomics analysis of Zur’s regulons was conducted in *Y. pseudotuberculosis*. The functions of Zur as a global regulator in metal homeostasis, motility, nutrient acquisition, glycan metabolism, and nucleotide metabolism, in turn, increasing the biofilm formation, stress resistance, and virulence were reviewed. The importance of Zur in environmental adaptation and pathogenicity of *Y. pseudotuberculosis* was emphasized.

## INTRODUCTION

Bacteria encounter diverse conditions in their living environment. To address these stresses, they modulate the expression of functional genes involved in metabolism *in vivo* through various regulators (1, 2). While many transcriptional regulators from different families have been identified, the functions and mechanisms of several regulators which essential for the fitness and survival of bacteria remain to be elucidated (3).

The Fur (ferric uptake regulator) superfamily is a significant group of regulators predominantly involved in metal homeostasis, stress defenses, and virulence in bacteria (4, 5). Regulators within the Fur superfamily, such as Fur, Zur, Mur, Nur, PerR, CatR, BosR, and Irr, play pivotal roles in different bacteria (6–12). Typically, bacteria harbor multiple members of the Fur superfamily. These regulators sense concentrations of metal ions like Fe^2+^, Zn^2+^, Mn^2+^, and Ni^2+^ and collaborate in metal regulation. They modulate the gene expression of metal import systems, efflux systems, and other proteins by binding to their promoter regions (4, 12). Among this superfamily, Fur and Zur have been most extensively studied across various species. Fur contains three Fe^2+^-binding sites: site 1 and 2 bind Fe^2+^ or Zn^2+^ to form dimer, sensing ion concentrations to bind DNA, while site 3 binds a [2Fe-2S] cluster to monitor intracellular iron levels (13, 14). Fur senses Fe^2+^ concentration and binds to the Fur-box in the promoter region of genes either activating or inhibiting transcription by interacting with RNA polymerase. Primarily, Fur suppresses the transcription of iron transporter genes like *fhuABCD*, *feoABC*, *fepABCD*, and *fecABCDE* to maintain iron balance (15, 16). Furthermore, Fur controls genes associated with oxidative stress resistance, antibiotics tolerance, motility, biofilm formation, and virulence of bacteria , establishing its role as a global regulator (12, 15). Similarly, Zur senses and maintains Zn^2+^ homeostasis *in vivo*, functioning with a structure and mechanism similar to Fur (17–19). Given the importance of zinc in cell growth, development, pathogen-host interaction, and stress resistance, the role of Zur in zinc homeostasis is vital for bacteria (20, 21). Zur’s function has been explored in over 20 species, with studies revealing its significant roles. Under nutrient-rich conditions, Zur decreases the transcription of zinc importer genes like *znuABC*, *zinT,* and *yciC*, while increasing the expression of zinc exporter genes such as *zitB*, *zrf,* and *zra* to ensure zinc equilibrium in bacteria and allow dynamic regulation as environments change (22–25). Additionally, Zur influences cell development, oxidative stress resistance, acid tolerance, antibiotic sensitivity, secretion systems, and virulence of bacteria (26–30). Although Zur has conserved functions, its role can vary across bacterial species (17). Thus, there is a pressing need to comprehensively study the precise functions of Zur in individual species.

*Y. pseudotuberculosis*, a congeneric species of *Yersinia pestis* and *Yersinia enterocolitica*, is an enteric pathogen responsible for a diverse range of gastrointestinal diseases. This bacterium has served as a model organism in the study of pathogenicity, virulence factors, and mechanism of environmental adaptation (31–35). In our previous research, both Fur and Zur were determined to have crucial roles in metal homeostasis, biofilm formation, stress resistance, and virulence of *Y. pseudotuberculosis* (30, 36, 37). Fur was shown to regulate not only aerobactin-mediated iron acquisition in an iron concentration-dependent manner but also T6SS4-mediated Mn^2+^ acquisition in response to manganese, compensating for the loss of Mur in *Y. pseudotuberculosis* (36). Concurrently, Zur exhibited dual functionality in zinc acquisition, both downregulating the expression of zinc transporter ZnuABC and upregulating T6SS4 in a Zn^2+^ concentration-dependent manner and in reaction to various stresses in *Y. pseudotuberculosis* (30). It has been established that Zur governs multiple functions in bacteria. The comprehensive regulatory functions of Zur in *Y. pseudotuberculosis* remain to be further explored.

In the present study, RNA-seq was employed to examine the transcriptional disparities between wild-type (WT) and Zur-deficient mutant strains, where the aim was to elucidate the overarching regulatory role of Zur in *Y. pseudotuberculosis*. Both quantitative real-time PCR (qRT-PCR) and electrophoretic mobility shift assay (EMSA) were conducted to validate the RNA-seq results. It was found that Zur regulons are involved in processes such as cation homeostasis, nutrient acquisition, motility, protein secretion, and multiple metabolisms. Additionally, Zur plays a role in biofilm formation and stress resistance. Collectively, this research identified Zur as a pivotal global transcriptional regulator instrumental in the environmental adaptation and pathogenicity of *Y. pseudotuberculosis*.

## RESULTS

### Genome-wide analysis of the genes regulated by Zur

**Fig 1:**
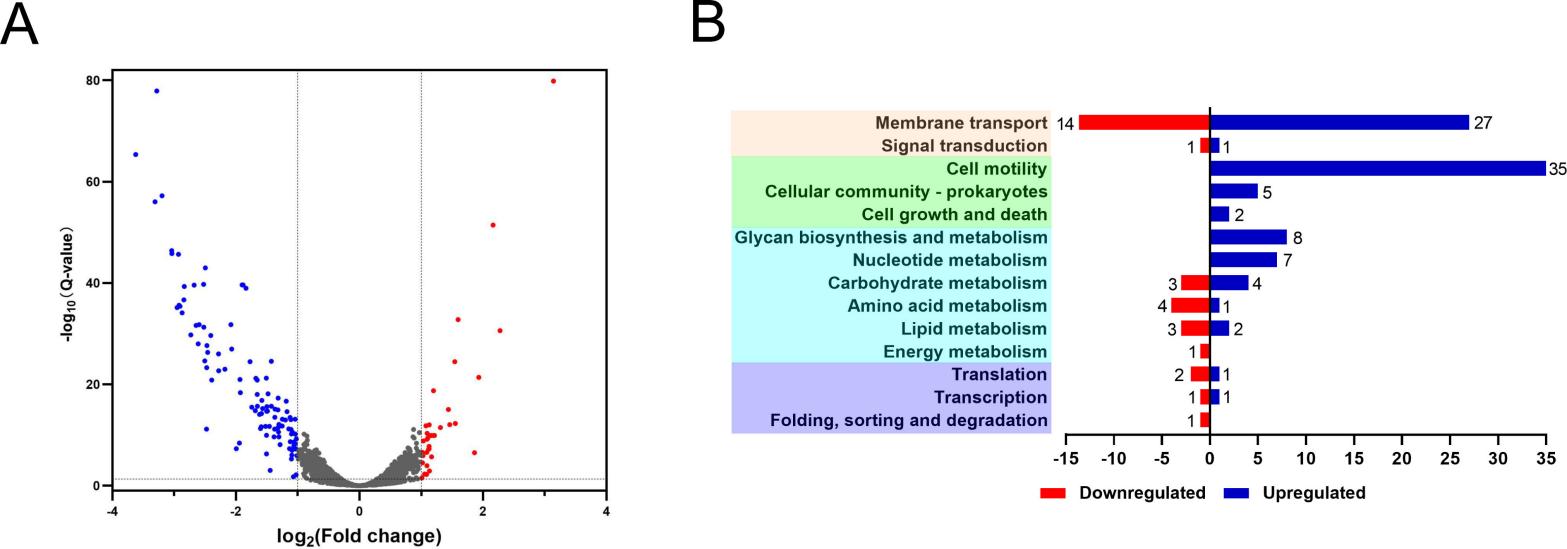
RNA-seq analysis of the genes regulated by Zur. (**A**) Volcano plot illustrating gene expression analysis, with the *x*-axis representing the log2(∆*zur*/WT) fold change in gene expression and the *y*-axis showing the log10(*Q*-value) for statistical significance. Downregulated genes are represented on the left side in blue, while upregulated genes are on the right side in red. Each treatment includes three biological replicates. (**B**) KEGG pathway enrichment analysis of DEGs between the ∆*zur* mutant and the WT strain.

In a preceding study, Zur was identified to downregulate the zinc transporter ZnuABC and concurrently upregulate T6SS4 (30), suggesting its potential multifunctionality. To delineate the comprehensive regulatory map of Zur, RNAs from both WT and ∆*zur* mutant strains in exponential phase were extracted and subjected to RNA-seq analysis (SRA accession: PRJNA1019653). The acquired data were subsequently processed, and the differentially expressed genes (DEGs) between the ∆*zur* mutant and the WT were identified at a *P*-value of <0.05 with a fold change >1.0. A total of 156 DEGs were identified: 110 DEGs were downregulated, and 46 were upregulated, in the ∆*zur* mutant based on the RNA-seq results (Table S2). Volcano plots and Kyoto Encyclopedia of Genes and Genomes (KEGG) pathway enrichment analysis results are illustrated in Fig. 1A and B. The DEGs were primarily clustered into 14 distinct pathways as per the KEGG pathway analysis, with membrane transport and cell motility emerging as the two predominant pathways.

Within the 41 DEGs classified under the membrane transport pathway, *znuA* (*ypk_2140*), *znuB* (*ypk_2142*), and *znuC* (*ypk_2141*) were observed to be downregulated by Zur with fold-changes of 3.67, 1.54, and 1.60, respectively. This finding aligns with previous studies (30). T6SS4 genes, as well as 15 genes associated with T6SS1-3 and Tat system, exhibited upregulation, albeit with less pronounced differences; the fold-changes were all <1.0 (Table S3). To validate this regulatory pattern, qRT-PCR was conducted (Fig. S1). The transcriptional levels of T6SS1-4 and Tat genes were upregulated by Zur, consistent with RNA-seq analysis. This suggests that Zur might activate the expression of T6SS1-3 and Tat system in a manner analogous to T6SS4.

### Zur regulates the expression of genes involved in metal homeostasis

Regulators from the Fur superfamily predominantly modulate the expression of genes related to metal homeostasis by sensing metal ion concentration *in vivo*. Structural analyses have shown that Zur in *S. Typhimurium* can bind Zn^2+^ or Co^2+^ in site 2 and that Zur in *Caulobacter crescentus* can cross-talk with Fur (38, 39). While Fur is known to regulate the uptake of Mn^2+^ in addition to Fe^2+^ in *Y. pseudotuberculosis* (36), it remains to be determined whether Zur can sense and regulate other metal ions in *Y. pseudotuberculosis*.

**Fig 2:**
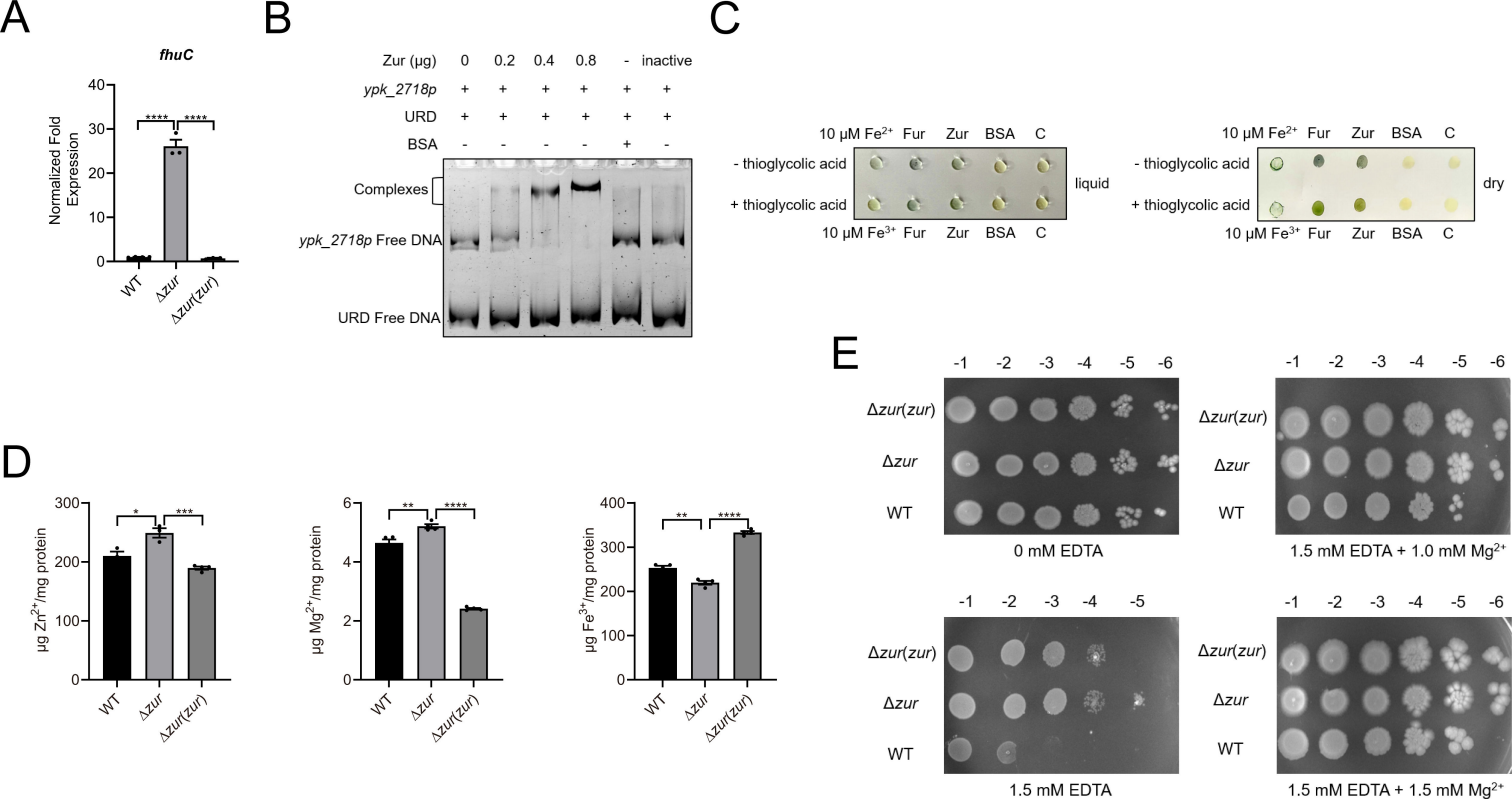
Zur regulates metal homeostasis. (**A**) qRT-PCR analysis of mRNA levels of *fhuC* (*ypk_2718*). (**B**) EMSA was performed to analyze the interactions between His6-Zur and *fhuB* promoter (*ypk_2718* p). Various amounts of Zur (0, 0.2, 0.4, and 0.8 µg), 50 ng DNA fragments, and URD (unrelated-DNA fragment) were used in each lane. (**C**) The iron-binding activity of Zur. C: control. (**D**) The intracellular metal ion concentrations of *Y. pseudotuberculosis* strains. Intracellular Zn^2+^, Mg^2+^, and Fe^3+^ were measured by inductively coupled plasma mass spectrometry in the *Y. pseudotuberculosis* WT, ∆*zur* mutant, and the complemented strain ∆*zur*(*zur*) grown to the end of the logarithmic phase in the Yersinia-Luria-Bertani (YLB) medium. (**E**) Tolerance of WT, ∆*zur* mutant, and the complemented strain ∆*zur*(*zur*) under metal-limited conditions were examined. Ten-fold dilutions of *Y. pseudotuberculosis* were spotted on YLB, supplemented with the indicated EDTA or metal ions concentrations. Plates were cultured at 26°C for 2 days. Data are shown as mean ± SEM (*n* = 3). *P < 0.05; **P < 0.01; ***P < 0.001; ****P < 0.0001.

Among the top-ranked DEGs from RNA-seq analysis, the putative Fe^3+^ transporter genes *fhuB* (*ypk_2719*), *fhuC* (*ypk_2718*), and *fhuD* (*ypk_2720*) were identified as being upregulated in ∆*zur* mutant, with fold-changes of 5.51, 6.68, and 8.81, respectively (Table S2). To verify the impact of Zur on *fhuC* transcription, qRT-PCR was conducted (Fig. 2A). The transcriptional level of *fhuC* in the ∆*zur* mutant increased by over 20-fold compared to the WT and the complemented strain. EMSA assays demonstrated that Zur binds directly to the promoter region of *fhuBCD* (Fig. 2B). Chromogenic reaction assays were utilized to test the ion-binding activity of Zur. With Fur serving as a control, Zur exhibited binding activity for both Fe^2+^ and Fe^3+^ (Fig. 2C). This suggests that Zur might regulate iron transporter FhuBCD by sensing the iron concentration. Subsequent analyses of intracellular metal ion concentrations of *Y. pseudotuberculosis* strains revealed significant increases in of Zn^2+^ and Mg^2+^ concentrations in the ∆*zur* mutant compared to the WT and the complemented strain (Fig. 2D). This aligns with observed upregulation of zinc transporter ZnuABC and magnesium transporter MgtE (*ypk_1341*, downregulated by Zur with a fold-change of 0.61 as indicated in Table S3) in ∆*zur* mutant in the RNA-seq data. However, iron concentrations decreased significantly in the ∆*zur* mutant. Several iron transporters exist in *Y. pseudotuberculosis*, including FeoABC, SitABCD, and IucABCD. Further examination of the RNA-seq data revealed three transporter genes (*ypk_2538*, *ypk _2751,* and *ypk _3892*) that were downregulated by Zur in mutant strain with fold-changes > 0.5 (Table S3). This could account for the decreased ion concentrations in the mutant. The growth assays under metal-limited conditions (by adding EDTA) demonstrated enhanced growth of *Y. pseudotuberculosis* in the absence of Zur when metal ions were scarce in the environment (Fig. 2E). In summary, Zur is pivotal in metal homeostasis in *Y. pseudotuberculosis*.

### Zur regulates genes involved in nutrient acquisition

**Fig 3:**
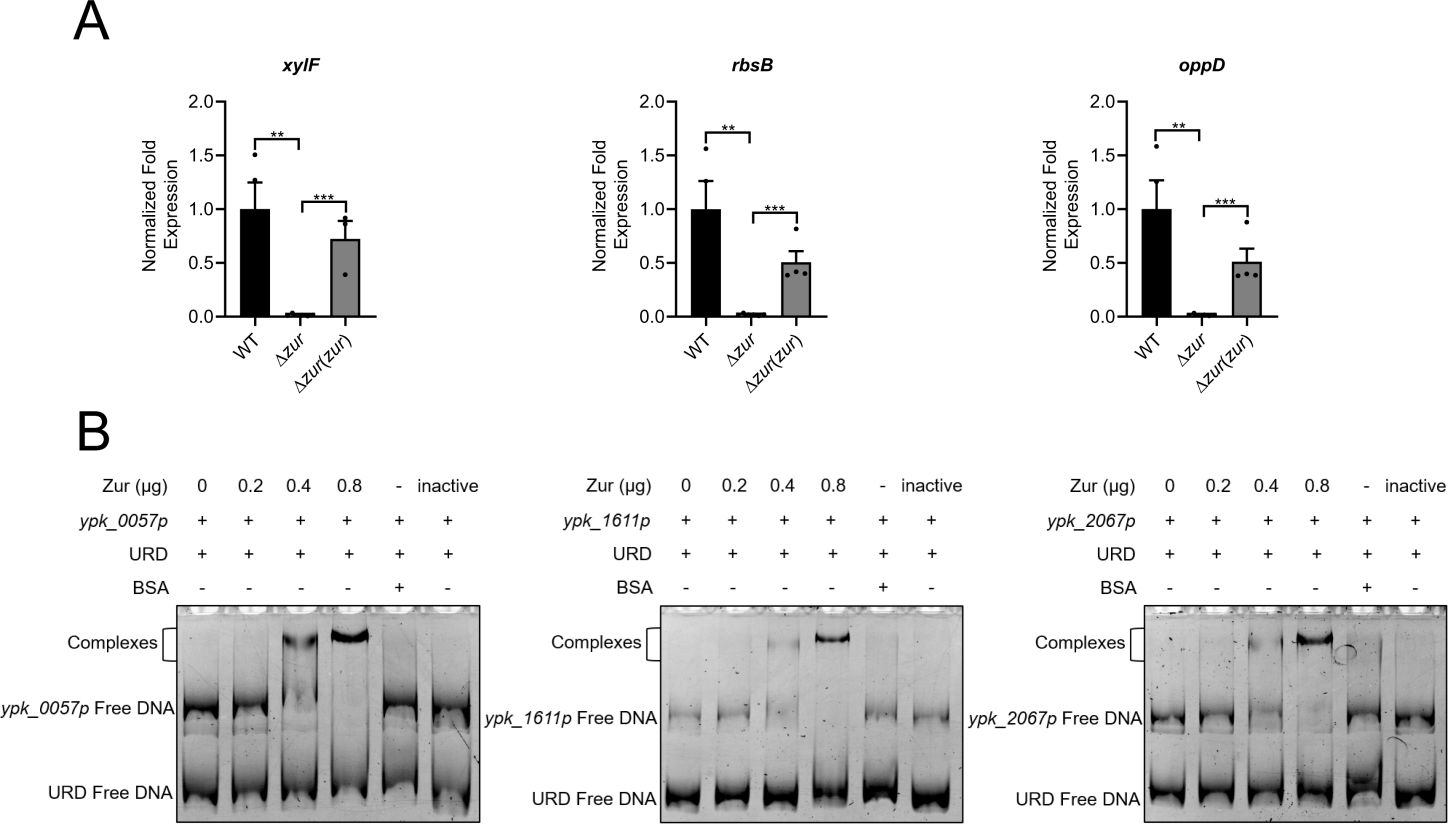
Zur upregulates nutrient transporter genes in *Y. pseudotuberculosis* by directly binding to its promoter. (**A**) qRT-PCR analysis of mRNA levels of *xylF* (*ypk_0057*), *rbsB* (*ypk_1611*), and *oppD* (*ypk_2067*). (**B**) EMSA was performed to analyze the interactions between His6-Zur and the promoters of nutrient transporter genes (*ypk_0057* p, *ypk_1611* p, and *ypk_2067* p). Various amounts of Zur (0, 0.2, 0.4, and 0.8 µg), 50 ng DNA fragments, and URD were used in each lane. Data are shown as mean ± SEM (*n* ≥ 3). **P < 0.01; ***P < 0.001.

Apart from the DEGs involved in metal ion homeostasis in the membrane transport group, several DEGs primarily encode for nutrient transporters, including D-xylose, ribose, erythritol, maltose, arginine, glutamate/aspartate, and oligopeptide transporters (Table S2). qRT-PCR and EMSA were conducted to verify the transcriptional regulation by Zur of *ypk_0057* (coding for D-xylose transporter, XylF), *ypk_1611* (coding for ribose transporter, RbsB), and *ypk_2067* (coding for oligopeptide transporter, OppD) (Fig. 3A and B). The data revealed that Zur directly upregulates the transcription of these genes by binding to their promoters. The modulation of these nutrient transporter genes suggests a potential role of Zur in nutrient acquisition in *Y. pseudotuberculosis*.

### Zur promotes motility by upregulating flagellar gene expression

**Fig 4:**
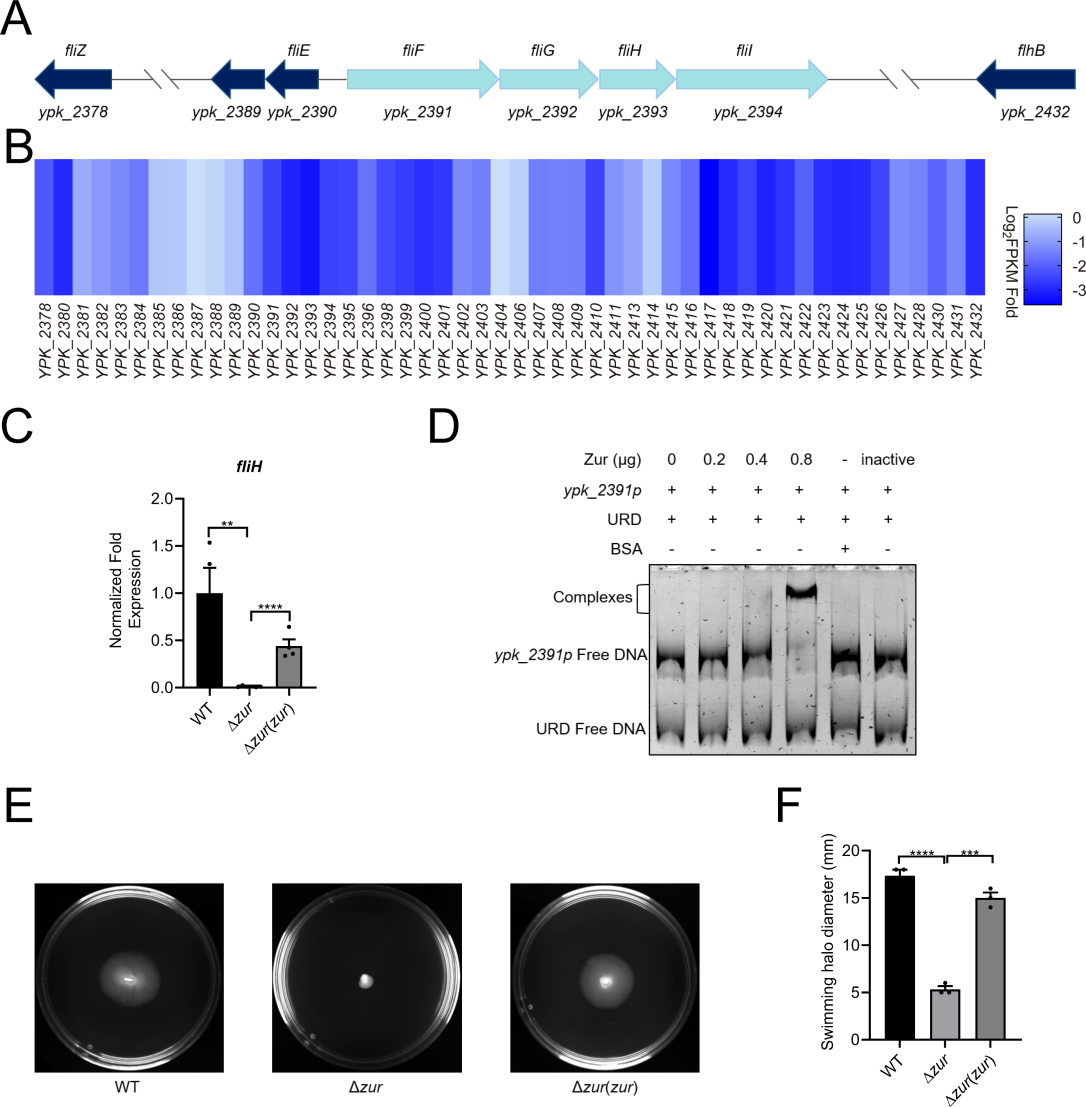
Zur promotes motility by upregulating the expression of flagellar genes. (**A**) Schematic illustration depicting the arrangement of flagellar genes within operons. (**B**) Heatmap of RNA-seq analysis. Heatmap was made by calculating log_2_(∆*zur* FPKM/WT FPKM). Different colors indicate different fold changes of gene expression. The darker the blue, the more significant the difference. FPKM, fragments per kilobase of transcript per Million mapped fragments. (**C**) qRT-PCR analysis of *fliH* (*ypk_2393*) mRNA levels. (**D**) EMSA was performed to analyze the interactions between His6-Zur and the promoters of flagellar-related genes (*ypk_2391* p). Various amounts of Zur (0, 0.2, 0.4, and 0.8 µg), 50 ng DNA fragments and URD were used in each lane. (E and F) Zur regulates the swimming motility of *Y. pseudotuberculosis*. Overnight cultures of WT, mutant, and the complementary strains were diluted with fresh YLB medium to an OD_600_ of 0.5 and spotted onto swimming plates (**C**), and the swimming halo diameters of each strain were measured (**F**). Data are shown as mean ± SEM (*n* ≥ 3). **P < 0.01; ***P < 0.001; ****P < 0.0001.

Both Fur and Zur have been observed to influence motility by regulating flagellar synthesis genes in *Escherichia coli* and *Pectobacterium odoriferum* (40, 41). In our study, the cell motility-associated DEGs showed the most significant group (35 DEGs) in the KEGG pathways regulated by Zur in *Y. pseudotuberculosis*. The arrangement of flagellar genes in operons is depicted in Fig. 4A, with a corresponding heat map analysis presented in Fig. 4B. Both qRT-PCR and EMSA verified that Zur regulates these operons by directly binding to their promoters (Fig. 4C and D). These DEGs encode nearly the entire flagellar apparatus, encompassing biosynthetic, component, assembly proteins, and regulators (FlgM, FliA, and FliZ). As depicted in Fig. S2, Zur may also indirectly influence the biosynthesis and assembly of flagellar system through these regulators. To assess the impact of Zur on motility, a swimming motility assay was performed using WT, ∆*zur* mutant and the complemented strains. The swimming motility of ∆*zur* mutant strain was notably reduced compared to the WT and the complemented strain (Fig. 4E and F). These findings suggest that Zur enhances the swimming motility of *Y. pseudotuberculosis* by upregulating the flagellar system.

### Zur participates in molecular pathways and energy metabolism in *Y. pseudotuberculosis*

**Fig 5:**
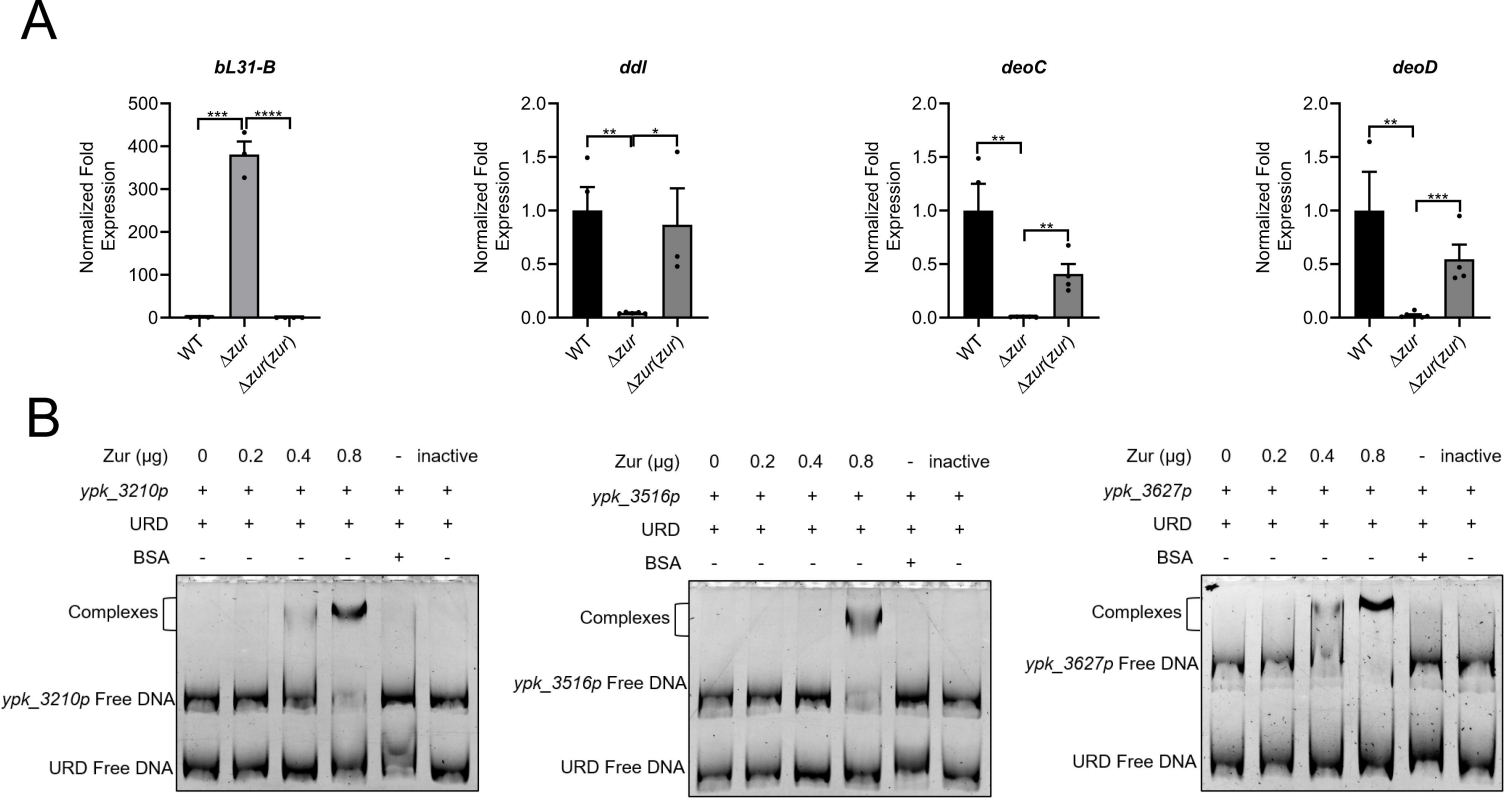
Zur participates in molecular pathways and energy metabolism in *Y. pseudotuberculosis*. (**A**) qRT-PCR analysis of mRNA levels of *bL31-B* (*ypk_3210*), ddl (*ypk_3616*), *deoD* (*ypk_3524*), and *deoC* (*ypk_3627*). (**B**) EMSA was performed to analyze the interactions between His6-Zur and various promoters (*ypk_3210*p, *ypk_3516*p, and *ypk_3627*p). Various amounts of Zur (0, 0.2, 0.4, and 0.8 µg), 50 ng DNA fragments, and URD were used in each lane. Data are shown as mean ± SEM (*n* ≥ 3). *P < 0.05; **P < 0.01; ***P < 0.001; ****P < 0.0001.

In addition to its influence on membrane transport and cell motility, Zur also regulates genes associated with the metabolism of sugar, lipid, amino acids, nucleotide, protein, and energy (Fig. 1B). qRT-PCR and EMSA were used to confirm these DEGs in various pathways (Fig. 5A and B). Zur was observed to positively regulate several enzymes, such as peptidoglycan biosynthesis-related D-alanine-D-alanine ligase Ddl (YPK_3516), purine-nucleoside phosphorylase DeoD (YPK_3624), and deoxyribose-phosphate aldolase DeoC (YPK_3627), by directly binding to their promoters. This underscores Zur’s regulatory influence on these molecular metabolic pathways. Moreover, Zur negatively regulated the large subunit ribosomal protein bL31-B (YPK_3210) and bL36-B (YPK_3211), which are implicated in both ribosome assembly during translation and zinc homeostasis (42, 43). The fact that Zur affects numerous metabolic pathways emphasizes its role as a significant global regulator in *Y. pseudotuberculosis*.

### Zur promotes biofilm formation and stresses resistance

**Fig 6:**
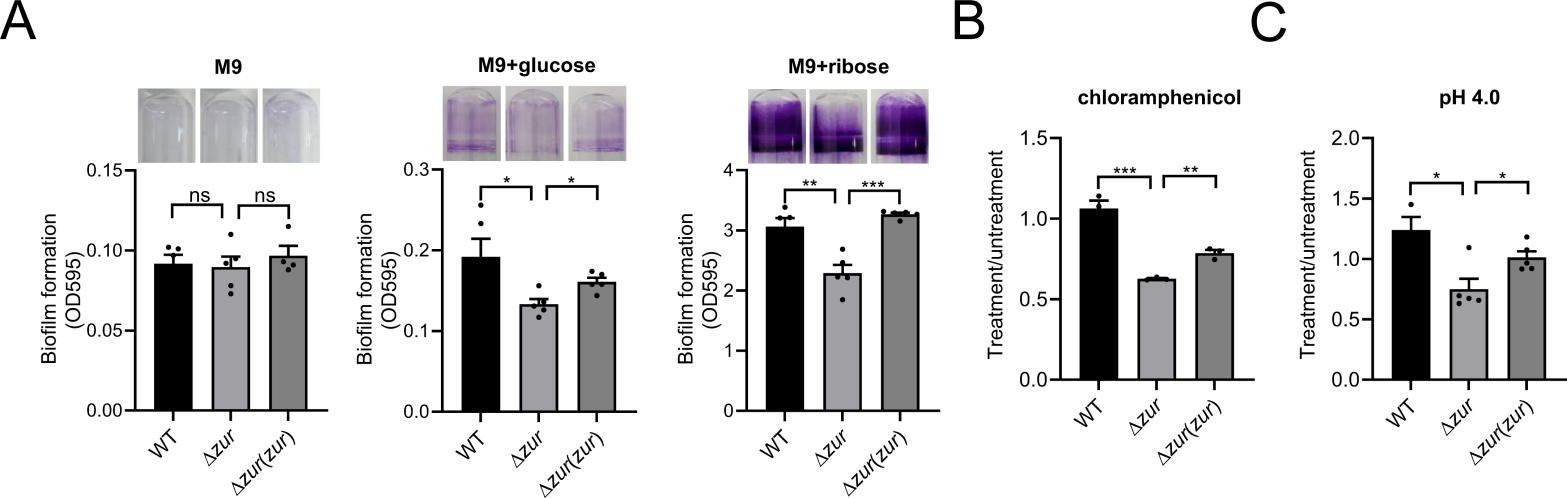
Zur promotes biofilm formation and stress resistance. (**A**) Effect of Zur on biofilm formation in M9 medium or supplemented with an additional monosaccharide. (B and C) Effect of Zur on stress resistance in *Y. pseudotuberculosis*. Survival rates of WT, ∆*zur* mutant, and the complemented strain ∆*zur*(*zur*) after challenging with 5 µg/mL chloramphenicol (**B**) or pH 4.0 (**C**) for 60 min were determined. Data are shown as mean ± SEM (*n* = 3). *P < 0.05; **P < 0.01; ***P < 0.001; n.s., not significant.

Given Zur’s regulatory activities across diverse metabolic processes, its influence on biofilm formation and stress resistance in various bacteria was explored (4, 44). To test Zur’s effect on biofilm formation, the biofilm of the WT, ∆*zur* mutant, and complemented strains were stained using crystal violet after 48 h of culture in M9 medium or supplemented with an additional monosaccharide. They were then quantified spectrophotometrically, as shown in Fig. 6A. The biofilm production in M9 medium was minimal, with no significant disparity between the WT and mutant strains. However, the introduction of glucose and ribose to the medium increased biofilm formation. Remarkably, the biofilm formation of the ∆*zur* mutant strain was markedly diminished compared to both the WT and the complemented strains, especially in ribose-supplemented medium. This could potentially be attributed to Zur’s positive regulation of monosaccharide transport and metabolism. Stress survival assays, involving exposure to chloramphenicol and acidic conditions, were also conducted (Fig. 6B and C). The survival rate of the ∆*zur* mutant strain showed a significant decrease under these stressors, suggesting that Zur enhances antibiotics and acid resistance in *Y. pseudotuberculosis*.

## DISCUSSION

Zur is a pivotal regulator with an influence that extends beyond merely maintaining zinc homeostasis. The global regulatory activities of Zur across over 20 species have been analyzed through RNA-seq, microarray and ChiP analyses (40, 41, 44–46). The suppression of zinc transporters, such as ZnuABC and ZinT, and the modulation of ribosomal proteins appear to be conserved across various bacteria (45). However, the exact roles of Zur can differ among species. Notably, within the *Yersinia* genus, the regulatory functions of Zur in *Y. pestis* differ from those in *Y. pseudotuberculosis*. Microarray expression studies identified 154 Zur-dependent genes of *Y. pestis*, comprising 90 upregulated genes and 64 downregulated genes. These genes are primarily involved in transporting, membrane protein synthesis, regulation, biosynthesis, and metabolism (47). Similar to its role in *Y. pseudotuberculosis*, Zur prominently regulates membrane transport proteins in *Y. pestis*, including those responsible for the uptake of zinc, iron, nickel, phosphate, amino acids, oligopeptide, and sugars. Furthermore, homologs of ribosomal proteins L31 and L36, as well as flagellar proteins, are subject to Zur’s regulatory effects in both species. However, Zur has a more pronounced effect on taurine, urease, and iron uptake proteins in *Y. pestis*. In contrast, genes linked to sugar transport and motility are subject to greater regulation by Zur in *Y. pseudotuberculosis* than in *Y. pestis*. Additionally, a higher number of DEGs associated with molecular and energy metabolisms are observed in *Y. pseudotuberculosis*. Collectively, these findings suggest that while Zur operates as a global regulator, its specific roles can vary, making its function in *Y. pseudotuberculosis* distinct from that in other bacteria.

Zur can regulate the expression of numerous divalent metal ion transporters and binding proteins, aiding *in vivo* homeostasis of cations such as zinc, iron, nickel, manganese, calcium, and cobalt (27, 29, 44, 48). In a prior study, Zur was demonstrated to downregulate the expression of zinc transporter gene *znuABC* by direct binding to its promoter using EMSA and β-Galactosidase analyses (30). RNA-seq analysis also revealed the downregulation of znuABC by Zur, with a subsequent increase in the zinc concentration in the ∆*zur* mutant strain, affirming its pivotal role in zinc uptake regulation. Contrary to the stable magnesium and iron concentration in the ∆*zur* mutant of *Cupriavidus metallidurans* (49), there were notable changes in the cation concentrations in the ∆*zur* mutant of *Y. pseudotuberculosis* (Fig. 2D). Eventhough the transcriptional change of magnesium transporter MgtE was modest (fold change of 0.6126), the magnesium concentration in mutant strain increased noticeably. This could be attributed to an accumulation of magnesium via the slightly upregulated transporters. Iron transport systems, such as Feo, Fec, Fep, Fbp, Fut, Sit, and Fhu, as well as aerobactin-mediated system, are orchestrated by both Fur and Zur, either directly or indirectly (37, 45, 50). In our research, only the ferric transporter FhuBCD was significantly downregulated by Zur in *Y. pseudotuberculosis* (Table S2). Contrasting with the increase of FhuBCD, the iron concentration dipped in the ∆*zur* mutant strain and spiked in the complementary strain (Fig. 2). This mirrors the regulatory role of Zur in iron as observed in *Xanthomonas oryzae pv. oryzae* (29). This phenomenon might stem from the function of other iron transport systems and role of the cross-talk between Zur and Fur in maintaining metal homeostasis. Notably, the absence of Zur in *Caulobater crescentus* increases the zinc concentration, leading to the suppression of Fur-regulated genes (39). Additionally, elevated zinc level *in vivo* also trigger ZitB expression, promoting iron export in *Streptomyces coelicolor* (24). Hence, Zur can mediate the regulation of iron-associated genes either directly or indirectly. However, it remains unclear whether Zur tailors these genes by sensing iron. Our findings reveal Zur’s iron-binding capacity *in vitro* (Fig. 2C), likely in the form of heme. Prior reports suggest that heme competes with Zn^2+^ for binding site 3 of Zur, altering its DNA binding activity in *Anabaena* sp. PCC7120 (51, 52). In *Salmonella Typhimurium*, Zur can bind cobalt at elevated concentration (38). The exact mechanism and implications of Zur’s potential iron or heme binding warrant detailed structural analysis.

Zur appears to facilitate the environmental adaptation and virulence of *Y. pseudotuberculosis* by elevating the expression of proteins pivotal for nutrient acquisition, chemotaxis and motility, cell adhesion, and virulence factors (Table S2). Beyond the regulation of metal ion transport, various nutrient transport systems, including the xylose (YPK_0057–0059), ribose (YPK_1161 and YPK_1162), erythritol (YPK_1961–1963), maltose (YPK_0378 and YPK_0382), simple sugar (YPK_2408–2411), glutamate/aspartate (YPK_3010), oligopeptide (YPK_2067 and YPK_2068), and nucleoside (YPK_1438 and YPK_3628) transport systems were upregulated by Zur. This suggests Zur’s role in nutrient acquisition in *Y. pseudotuberculosis*. Although similar regulation of amino acid and oligopeptide transport systems was revealed, Zur did not upregulate any sugar transporter in *Y. pestis* (47). The quorum-sensing molecule AI-2 transport system (YPK_3651–3653) and the purine-binding chemotaxis protein Chew (YPK_1750) were upregulated by Zur, aiding environmental signal sensing and motility. Chemotaxis and flagellar motility are crucial for functions such as swimming, biofilm formation, and virulence (53). While various bacteria, including *E. coli K-12*, *B. subtilis*, *C. metallidurans*, *P. odoriferum,* and *Y. pestis,* have Zur-influenced chemotaxis and flagellar genes, the prominence of its role differs (16, 27, 41, 47, 49). For instance, only three genes (*fliM*, *fliD,* and *flgD*) involved in chemotaxis and motility were revealed to be notably regulated by Zur in *Y. pestis* (47). But 35 flagellar genes, excluding AI-2 transporter and Chew genes, were notably upregulated by Zur in *Y. pseudotuberculosis*, suggesting Zur’s critical function in motility regulation. The swimming motility of the ∆*zur* mutant strain is markedly decreased in a swimming assay (Fig. 4E and F). Contrasting with *P. odoriferum,* where Zur reduces flagellar expression but does not alter motility in ∆*zur* (41), Zur in *Y. pseudotuberculosis* appears to have a wider regulatory impact.

Zur might influence acidic stress and antibiotics resistance through modulating membrane transporter, lipoprotein, cell wall plasticity, metabolism, and even biofilm formation (27, 54, 55). While biofilm formation is crucial for pathogen bacteria in terms of environmental adaptation and virulence, the mechanism of its formation and regulation remains enigmatic (56). Echoing findings in *Bacillus anthracis* and *Anabaena* sp. PCC7120, Zur in *Y. pseudotuberculosis* appears to promote biofilm formation (44, 54). The enhanced sugar acquisition, glycan biosynthesis, and transmembrane transport mediated by Zur in *Y. pseudotuberculosis* likely support the production of extracellular polysaccharides, which are fundamental for biofilm structure. The upregulation of peptidoglycan biosynthetic pathway including YPK_3516, YPK_3517, YPK_3518, and YPK_3520 (Fig. 5; Table S2) could facilitate the integrity of cell wall. Thus, by regulating aspects like metal uptake, nutrient acquisition, biofilm formation, cell wall biosynthesis, motility, virulence factors, filament, and T6SS (30), Zur could facilitate the stress resistance and pathogenicity of *Y. pseudotuberculosis*.

**Fig 7:**
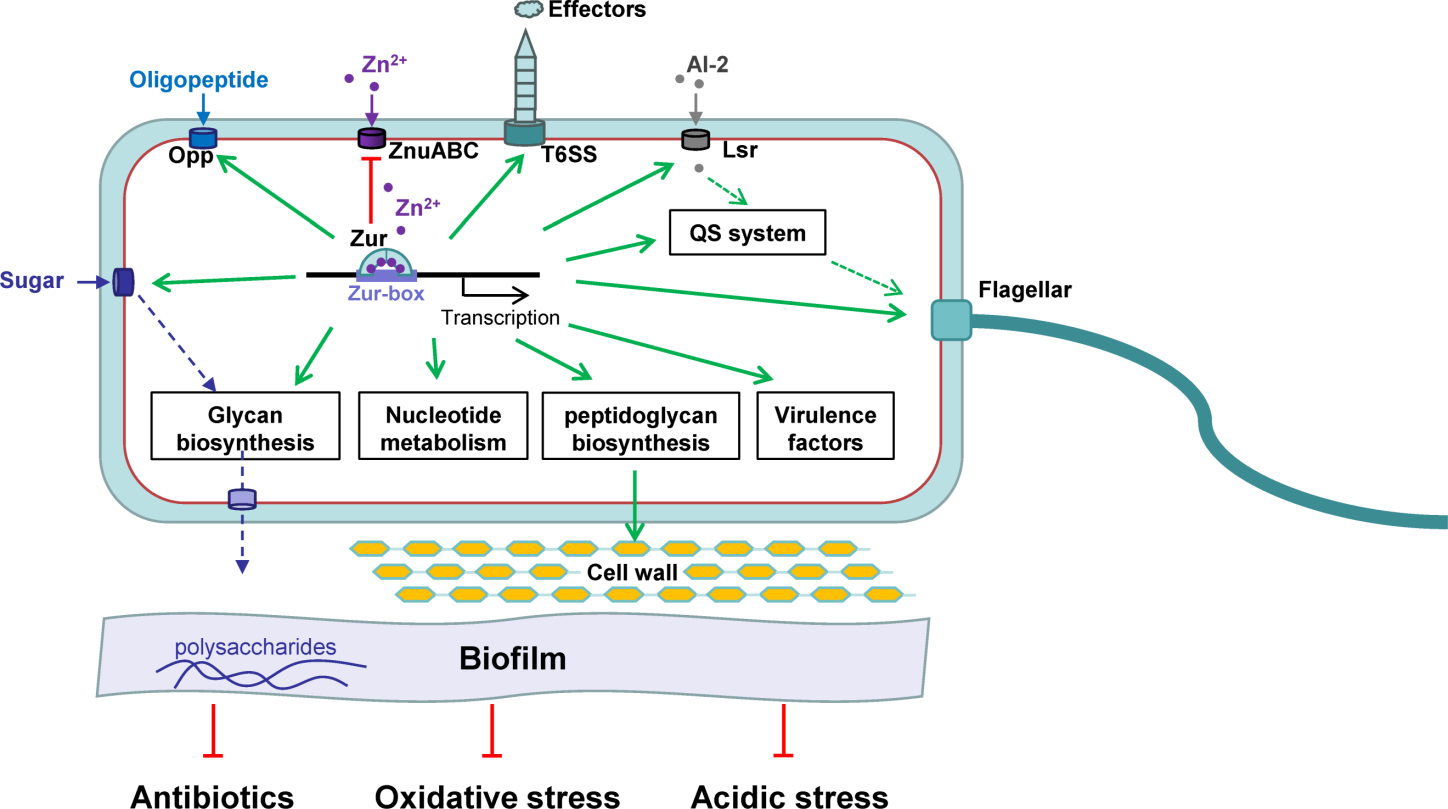
Functional versatility of Zur in metal homeostasis, motility, biofilm formation, and stress resistance in *Y. pseudotuberculosis*.

In conclusion, our study comprehensively analyzed the regulon of Zur in *Y. pseudotuberculosis* in this study. Beyond metal homeostasis, Zur is a global regulator influencing chemotaxis and motility, nutrient acquisition, glycan biosynthesis and metabolism, and nucleotide metabolism, in turn increasing stress resistance and virulence (Fig. 7). This research underscores Zur’s overarching regulatory capacity and significance for stresses resistance and pathogenicity in *Y. pseudotuberculosis*.

## MATERIALS AND METHODS

### Bacterial strains and growth conditions

The bacterial strains and plasmids utilized in this study can be found in Table S1. The growth conditions for the strains in this study were consistent with those employed in our previous research (30, 57). Briefly, *E. coli* was grown in Luria-Bertani (LB) medium at 37°C, supplemented with the necessary antibiotics. *Y. pseudotuberculosis* YPIII strains were cultured either in Yersinia-Luria-Bertani (YLB) broth (composed of 1% tryptone, 0.5% yeast extract, and 0.5% NaCl) or M9 medium (comprising 6 g/L Na_2_HPO_4_, 3 g/L KH_2_PO_4_, 0.5 g/L NaCl, 1 g/L NH_4_Cl, 1 mM MgSO_4_, 0.1 mM CaCl_2_, and 0.2% glucose) at 26°C, with appropriate antibiotics as needed. Specifically, nalidixic acid was added at a concentration of 15 µg/mL and kanamycin at 50 µg/mL.

### Overexpression and purification of recombinant protein

The purification of Zur was carried out in accordance with the procedures established in our prior study (30). In brief, the BL21(DE3) bacteria with the pET28a-*zur* plasmid were grown at 37°C in LB medium until they reached an OD_600_ of 0.5. Then, supplement with 0.5 mM IPTG induced at 24°C for 10 h. After harvesting, we disrupted the cells using sonication and purified the protein using His•Bind Ni-NTA resin (Novagen, Madison, WI). We confirmed the purified protein’s high purity (>95%) using SDS-PAGE analysis and measured its concentration using the Bradford assay.

### RNA-seq experiment

*Y. pseudotuberculosis* WT and ∆*zur* mutant strains were cultured in YLB medium to mid-logarithmic and normalized to OD_600_ = 0.8. Bacterial cells were harvested by centrifugation (each group set ≥3 replicates). RNA extraction, library construction, and RNA sequencing were commissioned by BGI Genomics (Shenzhen, China). According to the relative level of expression between the two groups of samples, the differentially expressed genes can be divided into up-regulated genes and down-regulated genes. For the samples with biological replicates, DESeq2 was used to conduct differential expression analysis among sample groups, and the differential expression gene set between the two biological conditions was obtained: KEGG (Kyoto Encyclopedia of Genes and Genomes) functional enrichment was performed on the selected DEGs, and genes were classified according to their functions to achieve the purpose of gene annotation and classification. When *P* value < 0.05, significant functional enrichment was considered.

### RNA extraction and quantitative real-time PCR

The *Y. pseudotuberculosis* strain was cultured in YLB medium in a shaker to OD_600_ = 1.0, and the cells were harvested by centrifugation. Total RNA was isolated using the *Steadypure* Universal RNA Extraction Kit AG21017 (Accurate Biotech, Hunan, China) following the manufacturer’s instructions. cDNA was synthesized by *Evo M-MLV* RT Reaction Mix Kit AG11728 (Accurate Biotech, Hunan, China). qRT-PCR was then performed using the SYBR Green *Pro Taq* HS Premix (Accurate Biotech, Hunan, China) in the LightCycler 96 thermal cycler (Roche, USA). The 16S rRNA gene served as a housekeeping gene to normalize mRNA abundance.

### Electrophoretic mobility shift assay

URD and DNA probes were amplified and purified on 1% agarose gel. URD was a 200 bp DNA amplified by qRT-PCR primers. Zur was incubated with 40 ng of non-specific URD and 40 ng of target promoter fragment in EMSA-binding buffer (10 mM Tris-HCl pH 7.5, 50 mM NaCl, 1 mM DTT, 500 mM KCl, 2.5% glycerol) at room temperature for 20 min (58, 59). The sample was then placed in a 6% native polyacrylamide gel in a 0.5 × Tris-borate-EDTA buffer at 100 V for 2 h at 4°C. The gel was stained with SYBR Safe DNA gel stain (Invitrogen, USA) and imaged with a fluorescent imaging system (Tanon 5200Multi, China) (60).

### Determination of intracellular ion content

The intracellular ion content was assessed following established procedures as described in prior research (37, 61). After reaching an optical density (OD_600_) of 1.0 through cultivation, the bacteria were harvested. Add the lysis solution at a ratio of 5 mL/g to resuspend the bacterial precipitation, and then lysis at 4°C overnight. Centrifuge at 10,000 rpm for 30 min, add the supernatant in a 1:100 ratio to a 2% chromatographic grade nitric acid solution, after treatment for 12 h, centrifuge at 10,000 rpm for 30 min, and then extract the supernatant. Samples were measured by inductively coupled plasma mass spectrometry, and relative intracellular ion content was calculated using protein concentration.

### Ion-binding assay

Before conducting the experiment, two specific staining solutions were prepared: one is composed of a mixture of 0.75 mM Ferene S and 15 mM thioglycolic acid added to 2% (vol/vol) acetic acid, and the other is a solution without 15 mM mercaptoacetic acid. Mix Fe^2+^, Fe^3+^, Fur, Zur, and BSA with two staining solutions, dot blot on the nitrocellulose membrane, and observe the droplet color (62, 63). In addition, the control group was mixed with dialysate and staining solution.

### Swimming motility assays

Overnight cultures of *Y. pseudotuberculosis* WT, mutant, and complementary strains were diluted with fresh YLB medium to an OD_600_ of 0.5, and 2 µL aliquots of the diluted cells were spotted onto 0.3% tryptone agar (1% tryptone, 0.5% NaCl, and 0.3% Bacto agar [Difco]) (60). Motility halos were measured after the plates were incubated at 26°C for 3 days.

### Biofilm formation assay

Biofilm formation was determined as described previously (60). In brief, bacterial cultures grown overnight were diluted 100-fold in 3 mL of fresh M9 medium (supplement with glucose or ribose), with the addition of appropriate antibiotics as needed. After 48 h of vertical incubation at 26°C with shaking at 150 rpm, the bacterial cultures were harvested following OD_600_ measurement, and the test tubes were subsequently washed twice with fresh M9 medium. Cells adhering to the inner surfaces of the test tubes were stained with 0.1% crystal violet for 30 min and subsequently washed twice with M9. The dye bound to the cells was eluted using 4 mL of 95% ethanol, and the absorbance of the eluted solution was quantified at 590 nm using a microplate reader.

### Stress survival assay

Take 1 mL of each bacterial solution (OD_600_ = 1.0) and centrifuge at 4,000 rpm for 3 min to collect the bacteria. After rinsing with M9 medium three times, transfer to pH 4.0 and normal M9 medium in a 1:50 ratio (64). After being stressed at 26°C, 150 rpm for 1 h, apply to YLB plates containing nalidixic acid and kanamycin, and incubate at 30°C for 24 h (37, 65). Chloramphenicol (5 µg/mL) stress was performed in the same manner as above. Count the colony-forming units (CFU) and calculate and analyze the survival rate. The survival rate is (CFU in the treatment group/CFU in the control group) ×100%.

### Statistical analysis

Statistical significance was performed using an unpaired two-tailed Student’s t-test with GraphPad Prism Software (GraphPad Software, San Diego, California, USA). Error bars represent ± SEM. *P < 0.05; **P < 0.01; ***P < 0.001; ****P < 0.0001; n.s., not significant.

## Data Availability

The data of RNA-seq were deposited in Sequence Read Archive (SRA) database (SRA accession: PRJNA1019653).

## SUPPLEMENTAL MATERIAL

Supplemental material spectrum.03756-23-s0001.docx Figures S1 and S2; Tables S1 to S3.

ASM does not own the copyrights to Supplemental Material that may be linked to, or accessed through, an article. The authors have granted ASM a non-exclusive, world-wide license to publish the Supplemental Material files. Please contact the corresponding author directly for reuse.
