# Supplementary material for: Functional versatility of Zur in metal homeostasis, motility, biofilm formation, and stress resistance in Yersinia pseudotuberculosis
Source: Microbiol Spectr. 2024 Mar 27;12(5):e03756-23. doi: 10.1128/spectrum.03756-23 (PMC11064496; doi:10.1128/spectrum.03756-23)
Supplement: Supplemental material — Figures S1 and S2; Tables S1 to S3. [file spectrum.03756-23-s0001.docx]

**Supplementary material**

**Functional versatility of Zur in metal homeostasis, motility, biofilm formation, and stress resistance in *Yersinia pseudotuberculosis***

**This file includes:**

Supplementary Figures 1

Supplementary Figures 2

Supplementary Tables 1

Supplementary Tables 2

Supplementary Tables 3


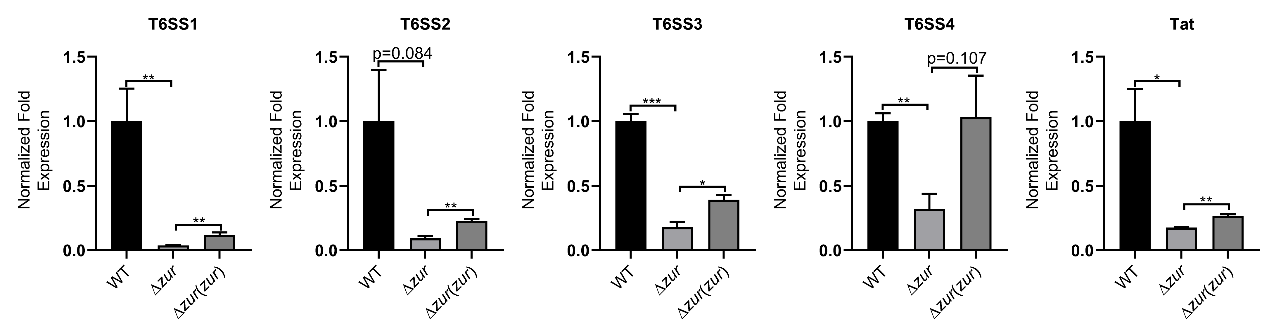


**Fig. S1. Zur regulates T6SS1-4 and Tat system.** qRT-PCR analysis of mRNA levels of T6SS1 (*ypk_0385*), T6SS2 (*ypk_0804*), T6SS3 (*ypk_1481*), T6SS4 (*ypk_3562*) and TatA system (*ypk_3942*). Mean values with standard deviations (error bars) from at least three repeats are shown. *p < 0.05; **p < 0.01; ***p < 0.001; ****, P<0.0001.


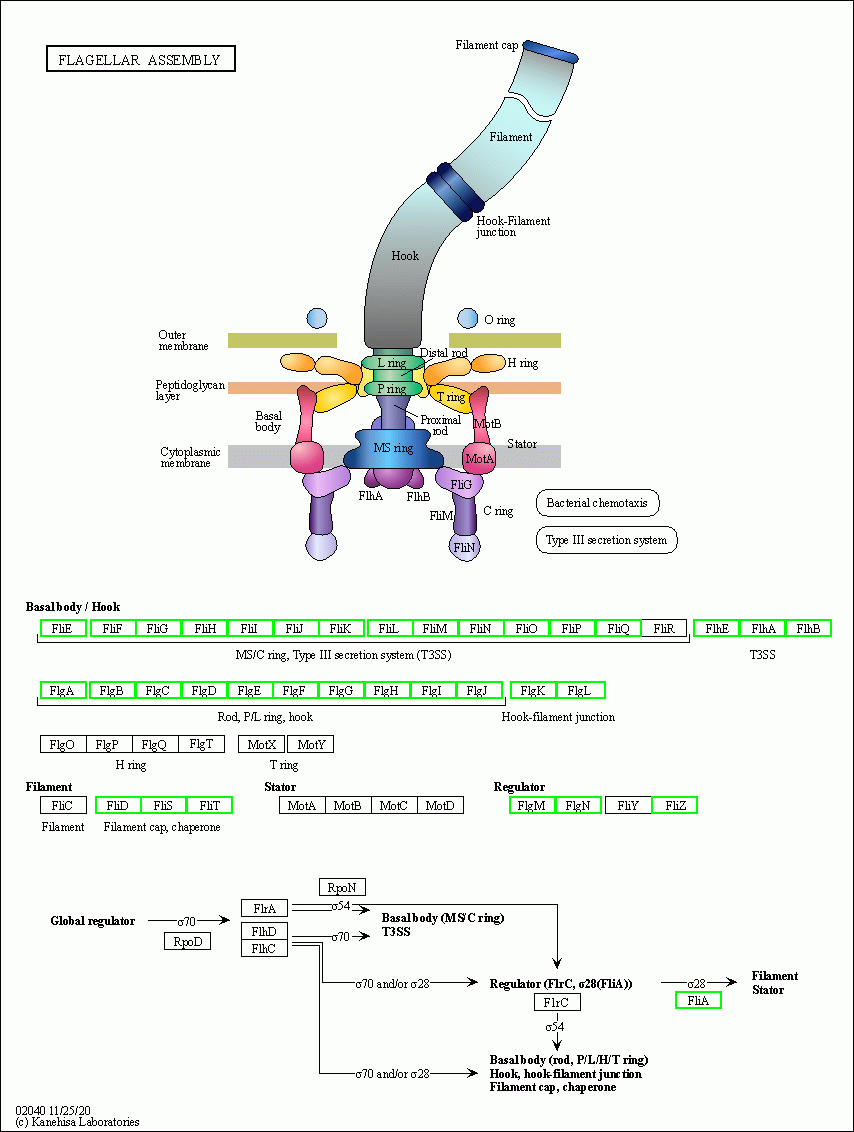


**Fig. S2. Zur regulates the biosynthesis and assembly of the flagellar system.** The schematics was produced by BGI Genomics (Shenzhen, China) according to the RNA-seq data. Genes in green boxes were identified as DEGs regulated by Zur in *Y. pseudotuberculosis*.

**Table S1.** Bacterial strains, plasmids and primers used in this study.

| **Strain, plasmid or primer** | **Relevant characteristics** | **Reference** |
| --- | --- | --- |
| ***E.coli*** |  |  |
| BL21(DE3) | Host for expression vector pET28a | Novagen |
| ***Y. pseudotuberculosis*** |  |  |
| WT | Wild-type *Y. pseudotuberculosis* containing pKT100, Nal^r^, Km^r^ | Cai et al.2021 |
| △*zur* | *zur* deleted in *Y. pseudotuberculosis* containing pKT100, Nal^r^, Km^r^ | Cai et al.2021 |
| △*zur*(*zur)* | △*zur* containing pKT100-*zur,* Nal^r^, Km^r^ | Cai et al.2021 |
| **Plasmids** |  |  |
| pKT100 | Cloning vector, p15A replicon, Km^r^ | Hu et al.2009 |
| pKT100-*zur* | *zur* in pKT100 for complementation | Cai et al.2021 |
| pET28a | Expression vector with N-terminal hexahistidine affinity tag，Km^r^ | Novagen |
| pET28a*-zur* | *zur* in pET28a for Zur protein expression and purification，Km^r^ | Cai et al.2021 |
| **Primer** | **5’-3’ sequence** | **Function** |
| *ypk_2719* F | CACAGTGCTTTTGCCAGATCCATAT | For EMSA |
| *ypk_2719* R | CTACTCAGTTTCCGCACGGTCATCA |  |
| *ypk_0057* F | GGCGGCAAACCGCAGATG |  |
| *ypk_0057* R | TGTTTTCTTTCCCCATAACTGCAGG |  |
| *ypk_1611* F | TTGCCAGTGATGCACCACTTTGTAT |  |
| *ypk_1611* R | ATTGTCTAATTAATTATTTCATGTAACAAAGAA |  |
| *ypk_2067* F | ATAACTCTGTCATTACTATTGATTAACAAAAA |  |
| *ypk_2067* R | GGTTGAACCCGAACGTTTAGTTAAG |  |
| *ypk_2390* F | TTGCTGTAAAGGACGCGATAATCCG |  |
| *ypk_2390* R | AGCACGCACGTATCACTCTTTTCAG |  |
| *ypk_3210* F | TGGAGATCCCGCCCAAGAT |  |
| *ypk-3210* R | AATGATTCCTTTTGGTATGTTAT |  |
| *ypk_3627* F | TTCTTGATACGAATAGCGTA |  |
| *ypk_3627* R | AAAACTCTCCAACACTGTTT |  |
| *ypk_3516* F | TAATTTAAGAGTTATCTGTCCGAAGAG |  |
| *ypk_3516* R | GGATATTTTAAGGGATGAAATAGAAA |  |
| ypk0395F | cgcattgcgtcagtcagcagga | For qRT-PCR |
| ypk0395R | ctctgtcgatgccagcaacca |  |
| ypk0804F | tcagcacacttattacgccgc |  |
| ypk0804R | taccgtgcgcggcaaagtatc |  |
| ypk1490F | gagcgtttactcgatgatgagcc |  |
| ypk1490R | ggcacggcataaagcccaac |  |
| ypk3562F | aagtctccgccatgaacacg |  |
| ypk3562R | gactcaaacgtggcgatgatcc |  |
| ypk3942F | gattatagcggtgatcgtcg |  |
| ypk3942R | ccctgttctttttcgtgactc |  |
| ypk2718F | caactgacgttaaagcacgtcg |  |
| ypk2718R | ggacacataggcgatgcgtg |  |
| ypk0057F | ttttactctccgcatgcgctg |  |
| ypk0057R | aaccaggacatcgacaccac |  |
| ypk1611F | aagccctgtcatttagcattgc |  |
| ypk1611R | tttgcaacatgtcttcgacgtc |  |
| ypk2067F | agatctgacggtgaccttcg |  |
| ypk2067R | cacggccattaaatttcgcag |  |
| ypk2393F | cgttgatgagcaacaggtgttg |  |
| ypk2393R | tcggtcaccataacttgccagtg |  |
| ypk3210F | atgaagcccaatatccaccctc |  |
| ypk3210R | accggtgtagtacggatgc |  |
| ypk3624F | ggcaacgccacatattaatgc |  |
| ypk3624R | ccatcacagaaatcttccggc |  |
| ypk3627F | atgaccgatttaaccgcctgc |  |
| ypk3627R | cgtaacgccttacgggcaac |  |
| ypk3516F | agaaagtcgcggtattgctg |  |
| ypk3516R | ccatgcagggcgataaacac |  |

Nal^r^, and Km^r^ represent resistance to naladixic acid and kanamycin at 20 and 50μg ml^-1^, respectively.

**References：**

Cai R, Gao F, Pan J, Hao X, Yu Z, Qu Y, Li J, Wang D, Wang Y, Shen X, Liu X, Yang Y. The transcriptional regulator Zur regulates the expression of ZnuABC and T6SS4 in response to stresses in *Yersinia pseudotuberculosis*. *Microbiological Research* 2021;249: 126787.

Hu, Y., Lu, P., Wang, Y., Ding, L., Atkinson, S., and Chen, S. OmpR positively regulates urease expression to enhance acid survival of *Yersinia pseudotuberculosis*. *Microbiology* 2009;155: 2522–2531.

**Table S2.** DEGs identified between the Δ*zur* mutant and the WT by RNA-seq in *Y. pseudotuberculosis*.

| **Functional group** | **Locus tag** | **Product** | **Kegg Orthology** | **log2(Δ*zur*/WT)** | **Qvalue(WT-vs-Δ*zur*)** | **Pvalue(WT-vs-Δ*zur*)** |
| --- | --- | --- | --- | --- | --- | --- |
| Cell growth and death | YPK_3515 | FtsQ | cell division protein FtsQ | -1.370913809 | 3.08E-14 | 5.95E-16 |
|  | YPK_3519 | FtsW | cell division protein FtsW | -1.32009321 | 1.06E-15 | 1.88E-17 |
| Cell motility | YPK_2378 | FliZ | regulator of sigma S factor FliZ | -2.178377042 | 1.06E-23 | 1.32E-25 |
|  | YPK_2380 | FliA | RNA polymerase sigma factor for flagellar operon FliA | -2.931338701 | 2.27E-46 | 1.17E-48 |
|  | YPK_2382 | FliD | flagellar hook-associated protein 2+ko02040//Flagellar assembly | -1.040071353 | 7.30E-14 | 1.44E-15 |
|  | YPK_2383 | FliS | flagellar protein FliS+ko02040//Flagellar assembly | -1.306129521 | 7.80E-13 | 1.63E-14 |
|  | YPK_2384 | FliT | flagellar protein FliT+ko02040//Flagellar assembly | -1.569268138 | 5.76E-16 | 9.76E-18 |
|  | YPK_2390 | FliE | flagellar hook-basal body complex protein FliE | -1.744170524 | 3.10E-16 | 5.18E-18 |
|  | YPK_2391 | FliF | flagellar M-ring protein FliF | -2.503542591 | 2.32E-25 | 2.62E-27 |
|  | YPK_2392 | FliG | flagellar motor switch protein FliG | -3.038908618 | 4.22E-47 | 1.99E-49 |
|  | YPK_2393 | FliH | flagellar assembly protein FliH | -3.310824251 | 9.41E-57 | 3.99E-59 |
|  | YPK_2394 | FliI | flagellum-specific ATP synthase | -2.520702391 | 5.01E-32 | 4.60E-34 |
|  | YPK_2395 | FliJ | flagellar FliJ protein | -2.473571021 | 4.95E-24 | 6.06E-26 |
|  | YPK_2396 | FliK | flagellar hook-length control protein FliK | -1.688192233 | 1.62E-15 | 2.90E-17 |
|  | YPK_2398 | FliL | flagellar basal body-associated protein FliL | -2.60957221 | 1.02E-28 | 1.03E-30 |
|  | YPK_2399 | FliM | flagellar motor switch protein FliM | -2.837381287 | 4.97E-40 | 3.28E-42 |
|  | YPK_2400 | FliN | flagellar protein FliN | -2.678063389 | 2.54E-40 | 1.61E-42 |
|  | YPK_2401 | FliO | flagellar biosynthetic protein FliO | -1.310067886 | 2.56E-11 | 6.33E-13 |
|  | YPK_2402 | FliP | flagellar biosynthetic protein FliP | -1.504731383 | 5.36E-07 | 2.31E-08 |
|  | YPK_2403 | FliQ | flagellar biosynthetic protein FliQ | -1.504731383 | 5.36E-07 | 2.31E-08 |
|  | YPK_2415 | FlgL | flagellar hook-associated protein 3 FlgL | -1.317617907 | 5.28E-18 | 8.08E-20 |
|  | YPK_2416 | FlgK | flagellar hook-associated protein 1 FlgK | -1.770784396 | 3.29E-25 | 3.87E-27 |
|  | YPK_2417 | FlgJ | flagellar protein FlgJ | -3.621842255 | 4.48E-66 | 1.69E-68 |
|  | YPK_2418 | FlgI | flagellar P-ring protein precursor FlgI | -2.921903424 | 2.45E-36 | 1.78E-38 |
|  | YPK_2419 | FlgH | flagellar L-ring protein precursor FlgH | -2.475771735 | 6.78E-12 | 1.63E-13 |
|  | YPK_2420 | FlgG | flagellar basal-body rod protein FlgG | -3.035888189 | 1.53E-46 | 7.59E-49 |
|  | YPK_2421 | FlgF | flagellar basal-body rod protein FlgF | -2.728644804 | 1.79E-30 | 1.73E-32 |
|  | YPK_2422 | FlgE | flagellar hook protein FlgE | -1.993015352 | 4.95E-08 | 1.67E-09 |
|  | YPK_2423 | FlgD | flagellar basal-body rod modification protein FlgD | -2.84141726 | 2.15E-37 | 1.52E-39 |
|  | YPK_2424 | FlgC | flagellar basal-body rod protein FlgC | -2.951625893 | 6.91E-36 | 5.37E-38 |
|  | YPK_2425 | FlgB | flagellar basal-body rod protein FlgB | -2.906348519 | 3.85E-36 | 2.90E-38 |
|  | YPK_2426 | FlgA | flagella basal body P-ring formation protein FlgA | -2.469478202 | 2.13E-28 | 2.21E-30 |
|  | YPK_2427 | FlgM | negative regulator of flagellin synthesis FlgM | -1.285011312 | 7.90E-09 | 2.53E-10 |
|  | YPK_2428 | FlgN | flagella synthesis protein FlgN | -1.426098402 | 2.12E-16 | 3.39E-18 |
|  | YPK_2430 | FlhE | flagellar protein FlhE | -1.934959198 | 1.08E-21 | 1.48E-23 |
|  | YPK_2431 | FlhA | flagellar biosynthesis protein FlhA | -1.368025942 | 7.69E-16 | 1.32E-17 |
|  | YPK_2432 | FlhB | flagellar biosynthetic protein FlhB | -2.870765625 | 7.96E-35 | 6.37E-37 |
| Cellular community - prokaryotes | YPK_3648 | LsrK | autoinducer-2 kinase/Quorum sensing | -1.585606543 | 5.94E-15 | 1.12E-16 |
|  | YPK_3649 | LsrR | lsr operon transcriptional repressor | -1.038108959 | 5.37E-08 | 1.88E-09 |
|  | YPK_3654 | LsrF | 3-hydroxy-5-phosphonooxypentane-2,4-dione thiolase | -2.596689104 | 1.58E-32 | 1.34E-34 |
|  | YPK_3655 | LsrG | (4S)-4-hydroxy-5-phosphonooxypentane-2,3-dione isomerase | -2.283390775 | 9.76E-27 | 1.08E-28 |
| Membrane transport | YPK_0238 | TsgA | MFS transporter, TsgA protein | -1.252154628 | 1.68E-12 | 3.68E-14 |
|  | YPK_0057 | XylF | D-xylose transport system substrate-binding protein/ABC transporters | -1.601678143 | 4.99E-12 | 1.16E-13 |
|  | YPK_0058 | XylG | D-xylose transport system ATP-binding protein/ABC transporters | -1.588503934 | 2.26E-12 | 5.12E-14 |
|  | YPK_0059 | XylH | D-xylose transport system permease protein/ABC transporters | -1.093269265 | 8.55E-07 | 3.78E-08 |
|  | YPK_1269 | HcaT | MFS transporter, PPP family, 3-phenylpropionic acid transporter | -1.102170041 | 5.98E-11 | 1.54E-12 |
|  | YPK_1438 | NupC | nucleoside transport protein | -1.836624765 | 1.04E-39 | 7.07E-42 |
|  | YPK_1611 | RbsB | ribose transport system substrate-binding protein/ABC transporters | -2.082051075 | 1.71E-32 | 1.49E-34 |
|  | YPK_1612 | RbsA | ribose transport system ATP-binding protein/ABC transporters | -1.119085475 | 2.08E-09 | 6.25E-11 |
|  | YPK_1618 |  | betaine/carnitine transporter, BCCT family | -1.373100906 | 6.47E-12 | 1.54E-13 |
|  | YPK_1961 | RbsA3 | erythritol transport system ATP-binding protein/ABC transporters | -1.035549191 | 5.37E-08 | 1.88E-09 |
|  | YPK_1962 | RbsC3 | erythritol transport system permease protein/ABC transporters | -1.381679284 | 2.26E-10 | 6.38E-12 |
|  | YPK_1963 | RbsB3 | erythritol transport system substrate-binding protein/ABC transporters | -1.653967586 | 1.39E-21 | 1.97E-23 |
|  | YPK_2408 |  | simple sugar transport system permease protein | -1.458499951 | 2.00E-12 | 4.46E-14 |
|  | YPK_2409 |  | simple sugar transport system ATP-binding protein | -1.479809146 | 7.60E-19 | 1.13E-20 |
|  | YPK_2410 |  | simple sugar transport system substrate-binding protein | -2.493702583 | 1.03E-43 | 5.58E-46 |
|  | YPK_2411 |  | simple sugar transport system permease protein | -1.130806089 | 5.04E-08 | 1.72E-09 |
|  | YPK_2067 | OppD | oligopeptide transport system ATP-binding protein/ABC transporters | -1.041758884 | 5.58E-09 | 1.75E-10 |
|  | YPK_2068 | OppC | oligopeptide transport system permease protein/ABC transporters | -1.03663065 | 4.22E-08 | 1.41E-09 |
|  | YPK_3010 | GltI | glutamate/aspartate transport system substrate-binding protein/ABC transporters | -1.012770289 | 1.10E-06 | 5.10E-08 |
|  | YPK_3219 | AmtB | ammonium transporter, Amt family | 1.128245823 | 9.24E-13 | 1.98E-14 |
|  | YPK_3628 | NupC | concentrative nucleoside transporter, CNT family | -1.884038279 | 2.36E-40 | 1.43E-42 |
|  | YPK_3651 | lsrC | AI-2 transport system permease protein/ABC transporters | -1.316684921 | 3.30E-12 | 7.58E-14 |
|  | YPK_3652 | lsrD | AI-2 transport system permease protein/ABC transporters | -1.522241413 | 1.88E-12 | 4.16E-14 |
|  | YPK_3653 | lsrB | AI-2 transport system substrate-binding protein/ABC transporters | -2.458526927 | 4.76E-27 | 5.16E-29 |
|  | YPK_0378 | MalE | maltose/maltodextrin transport system substrate-binding protein/ABC transporters | 1.084050957 | 0.00569378 | 0.0008873 |
|  | YPK_0382 |  | maltose operon periplasmic protein | 1.214740383 | 1.25E-10 | 3.42E-12 |
|  | YPK_1797 | KdgM | oligogalacturonate-specific porin family protein | -1.927607298 | 4.24E-19 | 6.18E-21 |
|  | YPK_1272 |  | nickel/cobalt transporter (NicO) family protein | 2.161822898 | 3.53E-52 | 1.58E-54 |
|  | YPK_2140 | ZnuA | zinc transport system substrate-binding protein/ABC transporters | 3.669382165 | 3.24E-163 | 6.86E-166 |
|  | YPK_2141 | ZnuC | zinc transport system ATP-binding protein/ABC transporters | 1.596321532 | 1.65E-33 | 1.36E-35 |
|  | YPK_2142 | ZnuB | zinc transport system permease protein/ABC transporters | 1.542135058 | 3.37E-25 | 4.05E-27 |
|  | YPK_2712 | ArtQ | arginine transport system permease protein/ABC transporters | 1.438435222 | 9.54E-16 | 1.66E-17 |
|  | YPK_2713 | ArtM | arginine transport system permease protein/ABC transporters | 3.141330253 | 1.46E-80 | 4.81E-83 |
|  | YPK_2715 |  | eamA-like transporter family protein | 4.594536244 | 2.31E-136 | 5.44E-139 |
|  | YPK_2718 | FhuC | iron complex transport system ATP-binding protein/ABC transporters | 6.683434906 | 2.01E-228 | 3.79E-231 |
|  | YPK_2719 | FhuB | iron complex transport system permease protein/ABC transporters | 5.513218343 | 6.21E-283 | 1.02E-285 |
|  | YPK_2720 | FhuD | periplasmic binding protein | 8.80727099 | 0 | 0 |
|  | YPK_2724 | ArtI | arginine transport system substrate-binding protein/ABC transporters | 1.017150925 | 2.47E-07 | 9.66E-09 |
|  | YPK_3658 | FrwC | PTS system, fructose-specific IIC-like component | -1.198893448 | 1.07E-13 | 2.19E-15 |
|  | YPK_3659 | FrwB | PTS system, fructose-specific IIB-like component | -1.617128217 | 8.43E-15 | 1.61E-16 |
|  | YPK_3660 | FrwD | PTS system, fructose-specific IIB-like component | -1.30557742 | 2.23E-10 | 6.19E-12 |
| Signal transduction | YPK_1750 | CheW | purine-binding chemotaxis protein CheW | -1.144545916 | 5.85E-12 | 1.38E-13 |
|  | YPK_1185 | RseC | sigma-E factor negative regulatory protein RseC | 1.002994155 | 3.74E-07 | 1.58E-08 |
| Folding, sorting and degradation | YPK_2898 | RhlE | ATP-dependent RNA helicase RhlE | 1.095877475 | 6.46E-10 | 1.89E-11 |
| Transcription | YPK_2780 | UxuR | GntR family transcriptional regulator, uxu operon transcriptional repressor | -1.10587249 | 7.74E-12 | 1.89E-13 |
|  | YPK_3220 | GlnK | nitrogen regulatory protein P-II 2 | 1.552250233 | 5.17E-13 | 1.07E-14 |
| Translation | YPK_4098 | bL31-A | large subunit ribosomal protein L31 | -1.425645075 | 2.69E-25 | 3.11E-27 |
|  | YPK_3210 | bL31-B | large subunit ribosomal protein L31 | 10.56745008 | 0 | 0 |
|  | YPK_3211 | bL36-B | large subunit ribosomal protein L36 | 9.3708952 | 0 | 0 |
| Amino acid metabolism | YPK_2458 | HpaD | 3,4-dihydroxyphenylacetate 2,3-dioxygenase | -1.016928049 | 6.26E-08 | 2.24E-09 |
|  | YPK_2716 |  | staphylopine/pseudopaline/yersinopine synthase | 6.570128319 | 0 | 0 |
|  | YPK_2717 |  | histidine 2-aminobutanoyltransferase | 6.858665664 | 0 | 0 |
|  | YPK_3001 | AsnB | asparagine synthase (glutamine-hydrolysing) | 1.162185937 | 1.23E-10 | 3.26E-12 |
|  | YPK_4189 | GlnA | glutamine synthetase | 1.196901038 | 1.87E-19 | 2.69E-21 |
| Carbohydrate metabolism | YPK_1922 | MtlK | D-arabinitol 4-dehydrogenase | -1.024304724 | 0.00677781 | 0.001085 |
|  | YPK_1974 | Orf6 | aldehyde dehydrogenase | -1.504992967 | 1.14E-10 | 3.00E-12 |
|  | YPK_3182 | Fcl | GDP-L-fucose synthase | -1.111570661 | 8.80E-14 | 1.78E-15 |
|  | YPK_3627 | DeoC | deoxyribose-phosphate aldolase | -3.354646021 | 1.59E-115 | 4.12E-118 |
|  | YPK_0494 | TreA | trehalose-6-phosphate hydrolase | 2.276828838 | 2.56E-31 | 2.41E-33 |
|  | YPK_0495 | TreB | PTS system, trehalose-specific IIB component | 1.862944177 | 2.97E-07 | 1.20E-08 |
|  | YPK_3846 | RhaA | L-rhamnose isomerase | 1.115469332 | 6.84E-08 | 2.47E-09 |
| Energy metabolism | YPK_2096 | GapA | glyceraldehyde 3-phosphate dehydrogenase | 1.036076702 | 1.44E-09 | 4.31E-11 |
| Glycan biosynthesis and metabolism | YPK_3180 | WbyQ | glycosyltransferase | -1.024953227 | 5.92E-08 | 2.09E-09 |
|  | YPK_3184 | WbyK | mannosyltransferase | -1.187103906 | 2.03E-17 | 3.21E-19 |
|  | YPK_3185 | Wzy | O-antigen biosynthesis protein Wxy | -1.49921461 | 2.35E-16 | 3.87E-18 |
|  | YPK_3186 | Wzx | LPS side chain defect: putative O-antigen transferase | -1.500906565 | 2.11E-15 | 3.88E-17 |
|  | YPK_3516 | Ddl | D-alanine-D-alanine ligase | -1.122004069 | 3.49E-14 | 6.82E-16 |
|  | YPK_3517 | MurC | UDP-N-acetylmuramate--alanine ligase | -1.018209506 | 7.05E-10 | 2.07E-11 |
|  | YPK_3518 | MurG |  | -1.49080368 | 1.82E-15 | 3.29E-17 |
|  | YPK_3520 | MurD | UDP-N-acetylmuramoylalanine--D-glutamate ligase | -1.081346767 | 2.91E-11 | 7.26E-13 |
| Lipid metabolism | YPK_3418 | DhaL | phosphoenolpyruvate---glycerone phosphotransferase subunit DhaL | -1.021946259 | 6.04E-10 | 1.75E-11 |
|  | YPK_3419 | DhaK | phosphoenolpyruvate---glycerone phosphotransferase subunit DhaK | -1.064127347 | 1.42E-08 | 4.56E-10 |
|  | YPK_3989 | GlpC | glycerol-3-phosphate dehydrogenase subunit C | 1.931012612 | 4.26E-22 | 5.51E-24 |
|  | YPK_3990 | GlpB | glycerol-3-phosphate dehydrogenase subunit B | 1.460715828 | 8.79E-13 | 1.86E-14 |
|  | YPK_4113 | GlpK | glycerol kinase | 1.075574558 | 1.49E-12 | 3.23E-14 |
| Nucleotide metabolism | YPK_2561 | Cdd | cytidine deaminase | -2.070002105 | 1.08E-27 | 1.15E-29 |
|  | YPK_3434 | CysC | adenylylsulfate kinase | -1.068532163 | 0.0164807 | 0.0031929 |
|  | YPK_3624 | DeoD | purine-nucleoside phosphorylase | -2.522278014 | 1.91E-40 | 1.08E-42 |
|  | YPK_3625 | DeoB | phosphopentomutase | -2.407586135 | 2.30E-30 | 2.27E-32 |
|  | YPK_3626 | DeoA | thymidine phosphorylase | -3.19629783 | 6.46E-58 | 2.58E-60 |
|  | YPK_3776 | CpdB | 2',3'-cyclic-nucleotide 2'-phosphodiesterase | -1.900629579 | 2.36E-40 | 1.45E-42 |
|  | YPK_3950 | Udp | uridine phosphorylase | -3.699240165 | 1.44E-113 | 4.07E-116 |
| No KO assigned | YPK_0255 |  | uncharacterized protein | 1.309732478 | 3.30E-12 | 7.62E-14 |
|  | YPK_0547 |  | protein of unknown function DUF883 ElaB | 1.105022413 | 4.05E-10 | 1.16E-11 |
|  | YPK_0699 |  | lipoprotein | -1.064686224 | 2.45E-09 | 7.44E-11 |
|  | YPK_1136 |  | urease accessory protein | 1.028202143 | 1.19E-06 | 5.50E-08 |
|  | YPK_1271 |  | protein of unknown function DUF1007/Periplasmic or exported protein | 3.103877745 | 1.20E-111 | 3.68E-114 |
|  | YPK_1445 |  | protein of unknown function DUF1479 | 1.095316226 | 4.60E-11 | 1.17E-12 |
|  | YPK_1522 |  | Fimbrial protein | 1.091445599 | 0.00011569 | 9.18E-06 |
|  | YPK_1619 |  | carnitine monooxygenase subunit | -1.581113845 | 1.43E-17 | 2.22E-19 |
|  | YPK_1676 |  | Antibiotic biosynthesis monooxygenase | 1.127489728 | 1.65E-08 | 5.36E-10 |
|  | YPK_1952 |  | putative virulence factor | -1.651517367 | 2.14E-16 | 3.47E-18 |
|  | YPK_1953 |  | putative virulence factor SrfB | -2.279170846 | 2.11E-23 | 2.68E-25 |
|  | YPK_1954 |  | Putative virulence factor | -2.645074664 | 2.38E-32 | 2.13E-34 |
|  | YPK_1960 |  | Predicted periplasmic lipoprotein | -1.101139993 | 5.26E-06 | 2.82E-07 |
|  | YPK_2407 |  | putative PfkB carbohydrate kinase family protein | -1.506665039 | 5.68E-22 | 7.49E-24 |
|  | YPK_2612 |  | conserved hypothetical protein | 1.0091702 | 0.0279554 | 0.0061991 |
|  | YPK_2694 |  | cold shock protein (beta-ribbon, CspA family) | -1.037179879 | 6.81E-11 | 1.78E-12 |
|  | YPK_2708 |  | putative lipoprotein | 1.089156096 | 2.54E-07 | 9.98E-09 |
|  | YPK_3002 |  | conserved hypothetical protein | 1.165887202 | 1.88E-06 | 8.99E-08 |
|  | YPK_3420 |  | short-chain dehydrogenase/reductase SDR | -1.090770755 | 9.57E-08 | 3.56E-09 |
|  | YPK_3623 |  | membrane protein | -1.247260018 | 7.40E-14 | 1.48E-15 |
|  | YPK_3631 |  | protein of unknown function DUF1328 | 1.190771809 | 1.25E-10 | 3.37E-12 |
|  | YPK_3632 |  | hyperosmotically inducible periplasmic protein | 1.126123326 | 5.30E-08 | 1.82E-09 |
|  | YPK_3949 |  | protein tyrosine/serine phosphatase | -1.678151142 | 6.22E-22 | 8.34E-24 |
|  | YPK_2781 |  | conserved hypothetical type 1 fimbrial protein | -3.28150303 | 1.25E-78 | 4.41E-81 |
|  | YPK_3675 |  | hypothetical protein | -1.654040015 | 9.42E-19 | 1.42E-20 |
|  | YPK_3859 | Zur | Fur family transcriptional regulator, zinc uptake regulator+ko02024//Quorum sensing | -9.276915464 | 0 | 0 |
| Unknown genes | YPK_RS11925 |  |  | -1.445727791 | 0.00089884 | 9.61E-05 |
|  | YPK_RS21735 |  |  | 1.054026331 | 0.0050311 | 0.0007639 |
|  | YPK_RS21865 |  |  | -1.171509142 | 2.43E-15 | 4.51E-17 |
|  | YPK_RS22040 |  |  | 1.020244483 | 2.90E-05 | 1.89E-06 |
|  | YPK_RS22045 |  |  | 1.133780768 | 0.00124351 | 0.0001393 |
|  | YPK_RS22230 |  |  | -1.942147597 | 3.63E-09 | 1.11E-10 |
|  | YPK_RS22700 |  |  | 1.139946124 | 1.24E-10 | 3.33E-12 |

**Table S3.** Differentially expressed genes involved in secretion system and ion transport with a fold change >0.3.

| ID | Gene ID | Gene Name | Kegg Orthology | log2(Δ*zur*/WT) | Qvalue(WT-vs-Δ*zur*) | Pvalue(WT-vs-Δ*zur*) |
| --- | --- | --- | --- | --- | --- | --- |
| T6SS1 | YPK_0385 | *hcp* | type VI secretion system secreted protein Hcp | 0.6511 | 3.41685E-05 | 2.27629E-06 |
|  | YPK_0390 | *impH* | type VI secretion system protein ImpH | -0.4687 | 0.023687154 | 0.005001737 |
|  | YPK_0391 | *impI* | type VI secretion system protein ImpI | -0.4165 | 0.047589661 | 0.012244898 |
|  | YPK_0392 | *vasD, lip* | type VI secretion system protein VasD | -0.7390 | 0.000433587 | 4.09295E-05 |
|  | YPK_0393 | *impJ* | type VI secretion system protein ImpJ | -0.5745 | 0.00090392 | 9.68182E-05 |
|  | YPK_0394 | *impK* | type VI secretion system protein ImpK | -0.5381 | 0.003052778 | 0.000412162 |
|  | YPK_0395 | *vasG, clpV* | type VI secretion system protein VasG | -0.5441 | 0.001203644 | 0.000133696 |
|  | YPK_0397 | *vasI* | type VI secretion system protein VasI | -0.4900 | 0.039437815 | 0.009618073 |
|  | YPK_0398 | *vasJ* | type VI secretion system protein VasJ | -0.3937 | 0.037107285 | 0.008944882 |
|  | YPK_0400 | *vasL* | type VI secretion system protein VasL | -0.4277 | 0.027850491 | 0.00616933 |
| T6SS2 | YPK_0804 | *vasG, clpV* | type VI secretion system protein VasG | -0.6372 | 0.018213651 | 0.00359946 |
|  | YPK_0809 | *vgrG* | type VI secretion system secreted protein VgrG | -0.8545 | 0.033036554 | 0.007644758 |
| T6SS3 | YPK_1481 | *hcp* | type VI secretion system secreted protein Hcp | -0.6176 | 0.007335512 | 0.001205981 |
|  | YPK_1490 | *impF* | type VI secretion system protein ImpF | -0.4732 | 0.0239422 | 0.005083772 |
| T6SS4 | YPK_3562 | *impF* | type VI secretion system protein ImpF | -0.4576 | 0.034231993 | 0.00800197 |
| Tat | YPK_3942 | *tatA* | Twin-arginine translocation (Tat) system | -0.7146 | 4.83E-05 | 3.37E-06 |
|  | YPK_2751 | *ABC.FEV.S* | iron complex transport system substrate-binding protein | -0.5007 | 0.036334053 | 0.008638746 |
|  | YPK_3892 | *ABC.FEV.P* | iron complex transport system permease protein | -0.5540 | 0.001419945 | 0.000164123 |
|  | YPK_2538 |  | IucA/IucC family protein, Ferric iron reductase FhuF-like transporter | -0.5441 | 0.166753704 | 0.061629782 |
|  | YPK_1341 | *mgtE* | magnesium transporter | 0.6126 | 0.00206133 | 0.000258152 |
